# Behavioral phenotype analysis highlights sleep heterogeneity and brain cellular activations in a mouse model of PTSD

**DOI:** 10.1016/j.ynstr.2026.100787

**Published:** 2026-02-23

**Authors:** Emma Lardant, Otilia Kelemen, Louise Pialoux, Coline Gervy, Blake Rea, Betty Poly, Damien Claverie, Frederic Chauveau

**Affiliations:** a Institut de Recherche Biomédicale des Armées (IRBA), Brétigny-sur-Orge, France; b École doctorale Biosigne, UMR-1195 INSERM, Bâtiment Grégory Pincus, Secteur, Le Kremlin Bicêtre, 94276, France; c Université Paris-Saclay, CNRS, Institut des Neurosciences Paris-Saclay, Saclay, 91400, France

**Keywords:** PTSD model, Behavioral phenotyping, Resilience, REM sleep, Cellular correlates, Amygdala, Periaqueductal gray matter

## Abstract

Exposure to intense and unavoidable stressor can lead to Post Traumatic Stress Disorder (PTSD) in some individuals. The heterogeneity of symptom expression, including sleep alterations, may explain the high rate of both non-responder and relapse of current treatments. Understanding individual-specific brain activity related to stress behavioral responses seems crucial for developing refined and personalized treatments strategies.

For this purpose, we evaluated the behavior of outbred mice through multiple tests up to 28 days after two inescapable electrical foot-shocks (FS). A two-step behavioral phenotype analysis successfully identified three phenotypes among FS mice. First, the scoring of behavioral alterations severity, based on PTSD-like symptoms, differentiated susceptible and resilient animals. Second, the specific type of stress-induced defensive behaviors further categorized two susceptible phenotypes: freezers and escapers.

Sleep patterns specific to phenotype emerged 14 days after the FS exposure. Notably, resilient mice exhibited more time spent in rapid eye movement sleep than susceptible animals, a variable that was negatively correlated to the behavioral alteration severity score.

Finally, behavioral profiling highlighted different c-Fos protein expressions in amygdala (AMG) and in the periaqueductal gray matter (PAG) across phenotypes, suggesting region-specific neural responses. Specifically, the severity of PTSD-like behaviors was correlated to the right lateral and central-lateral AMG cellular activations.

In conclusion, this study emphasizes the relevance of using composite score of multiple behavioral tests to better understand the complexity of stress responses and interindividual variability. Moreover, our findings suggest a role of REM sleep in promoting behavioral resilience to high-intensity stress.

## Highlights

• The behavioral alteration severity score of foot-shocked mice reveals resilient and two susceptible phenotypes.
• These phenotypes present different cellular activations in the amygdala and the periaqueductal gray matter.
• The behavioral profiling highlights specific sleep patterns between both resilient and susceptible mice.
• Behavioral alterations are correlated to both the activation of amygdala nuclei and rapid eye movement (REM) sleep.

## Graphical abstract

Decoding behavioral heterogeneity after foot-shock stress: resilience and susceptible phenotypes linked to REM sleep and brain cellular activations.The analysis of behavioral heterogeneity in mice receiving two electrical foot-shocks (FS; 1.6 mA, 2 s) highlighted three phenotypes with specific rapid eye movement (REM) sleep pattern and brain cellular activation within key brain regions implicated in PTSD (Amygdala, AMG, and periaqueductal gray matter, PAG). This heterogeneity was assessed by scoring (from 0 to 3), for each item, the deviation from the control (non-stressed) group median dataset of two variables for each animal. Then, each item is illustrated by a composite score (from 0 to 6) of those two variables.Four behavioral tests were chosen to model the four main PTSD-like symptoms: intrusion was assessed through percentage of freezing and escaping when animals were exposed to a FS cue reminder; the avoidance was quantified by the time spent and the velocity of animal in open arms of an elevated-plus maze; the altered reactivity was represented by the percentage of freezing and escaping when animals were exposed to a new context and new object; eventually the altered arousal was assessed in this study through the amplitude of the maximum response and the latency to this response during an airpuff startle test.Sleep was recorded during 24 h, 14 days after the foot-shock exposure. The two sleep stages slow-wave sleep (SWS) and rapid eye movement sleep (REM) were quantified every 5 sec-epoch. Sleep entry scores were calculated as the sum the latency to first sleep latency score and number of sleep episodes score. Sleep continuity scores were calculated as the sum of the scores of the percentage of time spent and the mean duration of sleep episodes for each sleep stage. The cellular activation variability was quantified by scoring the c-Fos cells numbers in AMG nuclei and PAG subcolumns. Two scores were assessed for each structure. For the amygdala, it is represented by the Basolateral (BLA) score, composite score of both basoanterior and lateral amygdala cellular activation scores, and the Central (CeA) score, composite of the central lateral and central medial amygdala nuclei activation scores. Both dorsal PAG (dPAG) and ventral PAG (vPAG) scores, composite scores of the cellular activation of the anterior and posterior part each PAG subcolumn, expressed the cellular activation of the PAG.The present results support the existence of resilient and two distinct susceptible (PTSD-like) phenotypes following foot-shock stress in mice and suggest a potential role of REM sleep in behavioral resilience. This study also underscores the relevance of using a composite score derived from multiple behavioral tests to better characterize complex interindividual variability.

**Figure:**
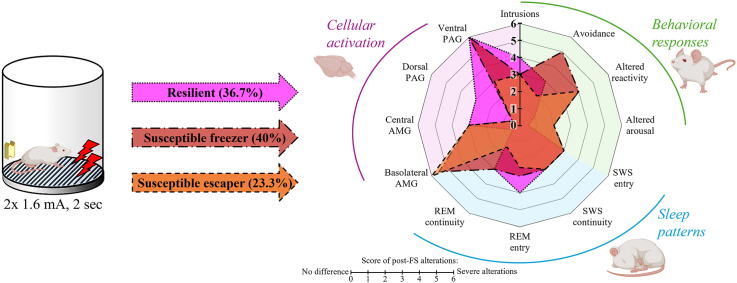

## 1 Introduction

Post-traumatic stress disorder (PTSD) is a neuropsychiatric condition that arises following exposure to a traumatic event. It is a complex pathology and a significant global health challenge. According to the World Mental Health Survey Consortium (Benjet et al., 2016), although more than 70% of the global population will experience at least one traumatic event in their lifetime, only about 4% will develop PTSD. However, treatment outcomes remain limited: many patients report no long-term improvement in symptom severity, and some even experience a worsening of symptoms or relapses (Asnis et al., 2004; Eftekhari et al., 2013; Wessa and Flor, 2007; Williams et al., 2022).

Understanding the mechanisms underlying PTSD development therefore represents a critical societal and clinical concern. PTSD symptoms are organized into four DSM-5 clusters (American Psychiatric Association, 2013; B to E), each containing several possible manifestations: B) Re-experiencing (intrusion) symptoms, C) avoidance of stimuli associated with the traumatic event(s), D) negative alterations in cognitions and mood associated with the traumatic event(s) and E) marked alterations in arousal and reactivity associated with the traumatic event(s). Patients may express only a subset of these symptoms, and their intensity varies across individuals (Gerger et al., 2014). Accordingly, several PTSD subtypes have been identified based on the severity of PTSD symptoms expression (Bryant, 2019; Campbell-Sills et al., 2022; Cloitre et al., 2013; Jongedijk et al., 2019).

Furthermore, insomnia and nightmares, part of the criterium E, are the most common complaints in PTSD patients, often accompanied by fragmented sleep, increased sleep latency, and reduced sleep efficiency (Lancel et al., 2021; Mellman et al., 2007; Pace-Schott et al., 2015; van Liempt, 2012; Weber and Wetter, 2022). Based on the Sleep to Forget, Sleep to Remember theory (SFSR; van der Helm and Walker, 2011; Walker and van der Helm, 2009), rapid eye movement sleep (REM) may strengthen the factual content of emotional memories (“remember”) while simultaneously reducing the associated emotional arousal (“forget”). This concept extends to the role of REM sleep in fear extinction (Murkar and De Koninck, 2018; Pace-Schott et al., 2009, 2015) and safety learning (Marshall et al., 2014; Ogeil and Baker, 2015). Nevertheless, studies examining alterations in REM sleep have yielded discrepant findings, with some reporting increased REM, while others report decreased or fragmented REM sleep (Kobayashi et al., 2007; Zhang et al., 2019). Such inconsistencies may be partly explained by the limited consideration of interindividual variability in PTSD symptoms expression, as well as by potential alterations in specific brain circuits regulating both fear processing and REM sleep.

Preclinical studies are essential to unravel the specific brain circuits underlying PTSD. Although several preclinical models of PTSD have been validated (Deslauriers et al., 2019; Verbitsky et al., 2020), recent research has shifted from simple stressed versus non-stressed comparisons toward deeper analyses of individual differences, distinguishing susceptible subjects (which develop persistent maladaptive symptoms without recovery) from resilient ones (which show adaptive responses and recover after trauma) (Colucci et al., 2020; Jeong et al., 2020; Sillivan et al., 2017; Toledano and Gisquet-Verrier, 2014; Torrisi et al., 2021). Those phenotypes, reflecting interindividual variabilities may underlie variations in brain activity, resulting from specific biological mechanisms.

Among potential biological mechanisms known to be crucial in the development of the PTSD (Nutt and Malizia, 2004), the four key anatomical-functional nuclei of the amygdala (AMG), the lateral, basolateral, central lateral and central medial amygdala, have been identified as the main structures involved in emotional processing and the formation of fearful memories (Ehrlich et al., 2009; LeDoux, 2003; Whittle et al., 2021; Wilensky et al., 2006). Furthermore, the key midbrain structure periaqueductal gray matter (PAG), is crucial for fear responses. Freezing (Frz) and escaping (Esc) are dimorphic innate defensive behaviors triggered by environmental threats and have been observed in both wild and laboratory animals (Blanchard et al., 1998; Tovote et al., 2015, 2016). The central nuclei of the AMG project to both dorsal and ventral columns of PAG, which are implicated in defensive behaviors (Carrive, 1993; Koutsikou et al., 2017; Linnman et al., 2012). Divergent pathways involving the amygdala (AMG) and periaqueductal gray (PAG) networks trigger these dimorphic defensive behaviors (Fadok et al., 2017; Kim et al., 2013; Shang et al., 2018) and thus may play a crucial role in interindividual variability following a traumatic event.

Previous studies have analyzed the heterogeneity of PTSD-like behavioral alterations among stress-exposed animals, highlighting the importance of individual behavioral profiling to better understand the neural basis of stress susceptibility and resilience (Ardi et al., 2016; Cohen et al., 2004; Richter-Levin et al., 2019). However, the variability of the behavioral stress response remains insufficiently integrated with biological and sleep correlates (Dopfel et al., 2019; Ritov et al., 2016; Wellman et al., 2016). To do so, we developed a phenotypic procedure using different behavioral tests on outbred Swiss mice over 28 days in a validated PTSD-like mouse model with electric foot-shocks (FS). Using a composite score of multiple behavioral tests assessing the severity of behavioral alterations, resilient animals were distinguished from susceptible animals. Then, the two main defensive behaviors, freezing and escaping, were found to define two distinct phenotypes of susceptible animals. Based on this phenotypic analysis, the longitudinal sleep analysis of the animals, as well as the long-term c-Fos protein expression analysis into the AMG and the PAG revealed specific sleep pattern and cellular expression for resilient and the two susceptible phenotypes. Those results highlight the crucial need to refine behavioral analyses in stress studies using composite score of multiple behavioral tests, which reveal neurobiological and sleep-related interindividual differences following a traumatic event. These results should stimulate further translational research to develop more personalized treatments for PTSD.

## 2 Materials and Methods

### 2.1 Animal care and use

Male Swiss mice (n = 47, 10–12 weeks; JANVIER Laboratories, France) were housed four per cage (21 ± 0.5 °C; 12 h light/dark cycle, lights on 10:00 a.m.) with ad libitum food and water, and socially isolated after foot-shock exposure. Behavioral testing was performed within the first 2 h of the light phase. Procedures complied with EU Directive 2010/63/EU and French law (Decree no. 2013-118) and were approved by the local ethics committee (project n° 502324 of 2020/02/20).

### 2.2 Behavioral procedures

#### 2.2.1 Behavioral test choice and order

**Fig. 1:**
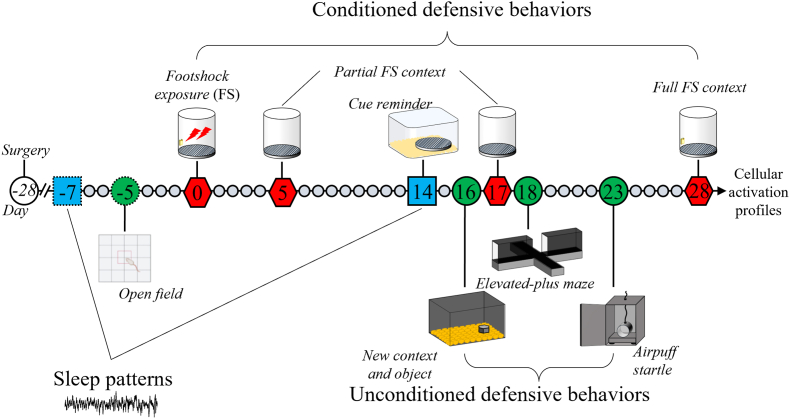
Experimental Procedure. Longitudinal behavioral study up to 28 days after exposure to two electric foot-shocks (FS, 1.6 mA, 2 s) in a specific and unique context (sponge, grid, diluted camphorated alcohol, Day 0). Each symbol represents a day; large ones stand for days with behavioral testing, before (dotted line) and after (plain line) FS. : Behavioral tests not associated with FS leading to the establishment a behavioral alteration severity index for each animal; : Behavioral tests associated with electric foot-shocks. Five days prior FS (−5): Open field test; Day 5 and Day 7: exposure to partial FS context; Day 16: neophobia tests (context and object); Day 18: elevated-plus maze; Day 23: air-puff startle response test and Day 28: re-exposure to full FS-associated context. : Polysomnographic recordings of animals' vigilance states over 24 h, 7 days before (−7) and 14 days after (14) exposure to electric foot-shocks. The animals were exposed 5 min to the grid before the sleep recording on day 14 (cue reminder).

Nine behavioral tests were conducted before and over 28 days post–foot-shock to generate a comprehensive behavioral profile (Fig. 1) at long-term delayed effect. Indeed, several works have shown that anxiety-like behavior gradually increased 10 days after stress exposure resulting from dendritic and functional changes in amygdala neurons (Chauveau et al., 2012; Mitra et al., 2005).

Tests were chosen to model PTSD-like behaviors in FS mice, reflecting core DSM-V symptom domains (Verbitsky et al., 2020):

● Intrusion: cue reminder exposure to assess foot-shocks conditioned responses.
● Avoidance: Elevated-Plus Maze (EPM) to assess anxiety-like behavior; reduced exploration of the open arms reflects avoidance of unprotected, brightly lit areas, paralleling avoidance behaviors observed in human PTSD. Altered arousal/reactivity: air-puff startle and exposure to a novel context and object to assess heightened startle and hypervigilance.
● Negative alterations in mood/cognition: anhedonia assessed via nest shape score (well-being indicator; Nollet, 2021)

To limit behavioral interference between tests, a single test was performed once a day. Order of non-conditioned test was from the less stressful (new context) to the more stressful test (startle). Conditioned responses were assessed at early (4 days post–foot shock exposure) and late (17- and 28-days post-exposure) time points to assess the strength of conditioned memory and its potential extinction.

#### 2.2.2 Foot-shocks exposure (FS, Day 0)

Mice were subjected to a validated PTSD-like foot-shock protocol (Philbert et al., 2011; Siegmund and Wotjak, 2007). Mice were randomly assigned to foot-shock (FS, n = 31) or no foot-shock control (No-FS, n = 16) groups. All 47 animals were placed individually in a transparent Plexiglas cylindrical chamber (24.5 × 35 cm; iMETRONIC®, France) for 330 s with a camphorated alcohol–soaked sponge. After 120 s of exploration, two foot-shocks (2 s, 1.6 mA, 30 s interval) were delivered only to FS animals while No-FS animals remained in the chamber for the same duration without receiving shocks. Mice remained in the chamber for an additional 120 s. All animals were singly housed following exposure until study completion.

#### 2.2.3 Cue reminder test (day 14, D14)

On day 14 (D14), the FS metallic grid was placed for 5 min in the sleep recording cage of both FS and No-FS animals. Behavior was recorded and analyzed using EthoVision XT16 (Noldus, the Netherlands, Noldus et al., 2001).

#### 2.2.4 Partial/full context re-exposure (D5, D17 and D28)

Animals were re-exposed to the foot-shock chamber for 5 min without shocks. Sessions on days 5 and 17 were conducted without the alcohol-soaked sponge (partial context), while day 28 included the full context (box, grid, sponge). Animals were perfused 90 min after the completion of the D28 test.

#### 2.2.5 New object/new context test (Day 16)

Mice underwent a 10-min session with two 5-min trials. In trial 1, animals explored a novel context (34x27 × 22 cm, glass box with fresh bedding material at floor; <20 lux in the center); after 2 min in the home cage, trial 2 consisted of re-exposure to the context with a novel salient object (an 80 × 80 mm wooden block cleaned with a solution of 10% diluted peach essential oil). Behavior was manually scored from video using Solomon Coder (Beta 19.08.02, Milan, Italy).

#### 2.2.6 Elevated-plus maze (D18)

Each mouse was placed into the center of the Elevated-plus-maze, made of black Plexiglas (30 × 5 cm in each 4 arms, two without walls and two enclosed by 15 cm high walls; 60 lux in the open arms and 20 lux in the closed arms). The maze was 40 cm above ground. Spontaneous exploration was assessed in an elevated-plus maze over 5 min. The maze was cleaned with disinfectant spray between trials. Behavior was recorded and analyzed using EthoVision XT16.

#### 2.2.7 Air puff startle test (D23)

Mice were placed in an acrylic cylinder within the SR-LAB Startle Response System (San Diego Instruments, CA, USA). Sessions included 5 min habituation followed by 40 trials, each comprising a 100 ms white noise burst and 20 ms air puff. Maximum response amplitude (peak) and latency-to-peak were recorded; trials with pre-stimulus movement were excluded. Habituation was quantified as the ratio of the median of the last five trials to the median of the first five trials for both variables. Hyperarousal was defined as impaired habituation and reduced response latency.

### 2.3 Composite score assessment

To quantify behavioral alterations after foot-shocks, a composite score was calculated by summing the score of eight variables from four different behavioral tests. The score was adapted from the PTSD Checklist for DSM-5 (PCL-5), which screens for PTSD and quantifies symptom severity according to DSM-5 criteria (Blevins et al., 2015).

#### 2.3.1 Variables selection

Variables were selected based on variance and literature prevalence (Verbitsky et al., 2020) following three steps: (1) confirm common use in prior studies (2) rank by standard deviation; (3) remove redundant measures; Two additional variables captured the full freeze–flight–fight continuum: freezing, defined as complete immobility excluding respiration (>3 s), and escape behaviors, encompassing inter-individual variability from wall climbing to low- and high-amplitude jumps.

#### 2.3.2 Score assignment to variable values

**Fig. 2:**
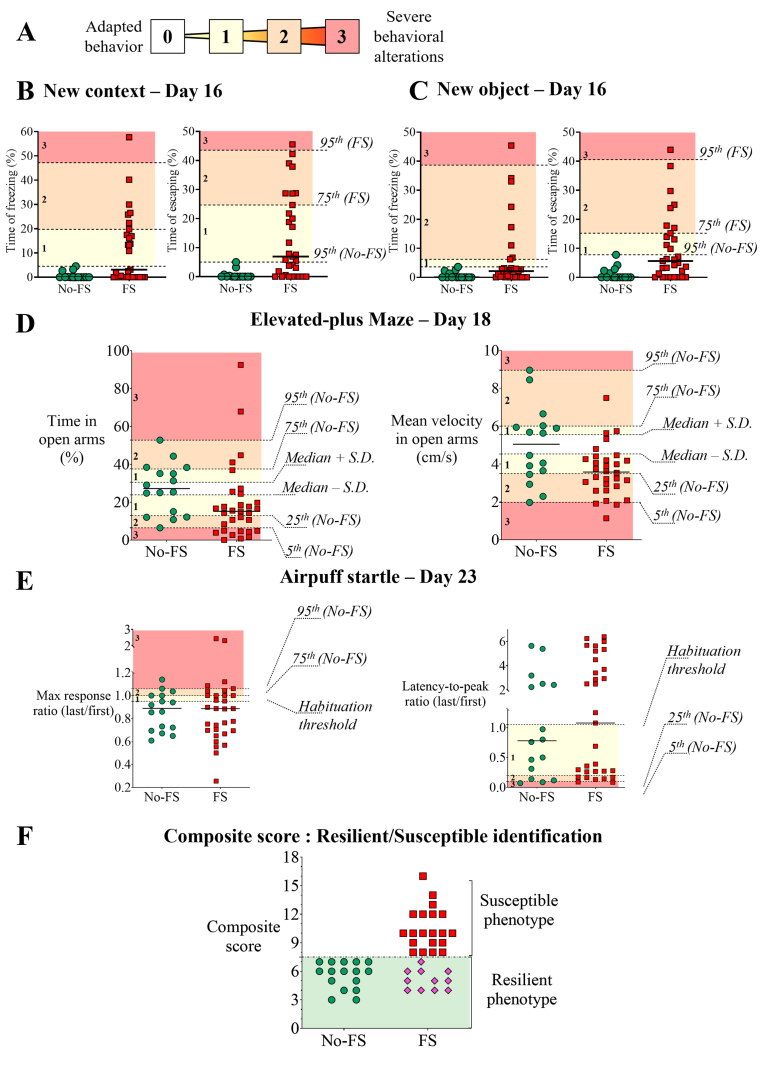
First step of behavioral phenotypes analysis and characterization of resilient and susceptible phenotype in foot-shocked (FS) mice. **A**. Attribution to a 4-point-score (0 to 3) to each individual for each variable, according to the level of the dispersal of the behavior from the no foot-shocked (FS) control group data set median. Those scored characterized the behavioral responses of animals during each test: from “adapted,” scored as 0 to “severely altered”, scored as 3. Two variables were selected for each test (B-E). **B-C**. Percentage of freezing and percentage escaping on day 16 during the new context (B) and the new object (C) exposure. **D**. Percentage of time spent (left) and the mean velocity (right) on open arms of the Elevated-plus Maze (EPM; day 18). **E**. Ratio of the median of maximum amplitude of response of the 5 last trials over the 5 first trials (Max response ratio, left) and the ratio of the latency to obtain the maximum amplitude (Latency-to-peak ratio, right) during the airpuff startle. A mouse was considered habituated when the ratios were below (left) or above (right) 1. **F.** The first step of the classification leads to a composite score for each mouse, above a score of 7 (maximal score for no foot-shocked control animals), the foot-shocked mice's phenotype is considered as susceptible phenotype, a score below 7 is considered resilient phenotype. For B-E: Color-coded score from 0 to 3 (0, 1, 2 and 3; numbers on the left; each symbol represents one animal). □ FS exposed mice, n = 31, ໐ No-FS (controls) mice, n = 16, bar: median). Score boundaries (dotted lines) were defined using the median, standard deviation (S.D.), and the 25th, 75th, and 95th percentiles of the No-FS dataset. For novel context and object exposure (B), control values were too low to serve as a reference; therefore, the maximum of the No-FS dataset and the 75th and 95th percentiles of the FS dataset were used instead.

Behavioral alterations were quantified using a four-point score (from 0 to 3) applied to each selected variable in FS and control groups (Fig. 2A). This score provides sufficient granularity to distinguish severity levels while minimizing complexity. Thresholds for each score were defined based on the statistical distribution of the No-FS group to ensure a standard, transparent, and reproducible framework, using the median, 25th, 75th, and 5th/95th percentiles (with an exception for the new context and object exposures). This percentile-based, non-parametric approach provides a data-driven framework to classify behavioral alterations from moderate to severely atypical responses.

For the new context and object tests (Fig. 2B and C), percentages of freezing and escape were used, FS behavior was considered adaptive if within the No-FS range. Score attribution was not restricted to control values, as these were nearly null and therefore unsuitable as a reference. Instead, the values selected were the 95th percentile of the No-FS dataset (integer 0), followed by the 75th and 95th percentiles of the FS dataset (integers 1-3).

For the elevated-plus maze (Fig. 2D), anxiety-like behavior was assessed via time and velocity in open arms, with opposing behaviors (risk-taking vs. anxiety) scored in opposite directions relative to integer 0; score thresholds were based on No-FS 5th, 25th, 75th and 95th percentiles.

For the startle response test (Fig. 2E), habituation was quantified using ratios calculated as the median value of the last five trials divided by the median value of the first five trials of a 40-trial session, for both the maximum response amplitude and the latency to peak. Hyperarousal was defined as reduced habituation, reflected by a maximum response amplitude ratio ≥0.95, and by shortened latency, reflected by a latency-to-peak ratio ≤1.05. Thresholds corresponding to scores of 2 and 3 were determined based on the distribution of the data (median, 25th percentile and 75th percentile values) of the No-FS dataset.

The composite score (Fig. 2F), summing these values across all tests, provides a single quantitative measure of the severity of behavioral alterations for each individual.

#### 2.3.3 Radar plot of the behavioral alteration scores (Fig. 4F)

The radar plots represent the median scores for intrusions, avoidance, altered reactivity, and altered arousal, based on the variables used to calculate the composite scores. The intrusions score combines the scores for the percentage of freezing and percentage of escaping when animals were exposed to the cue reminder (metallic grid) at D14. The avoidance score is the sum of the scores for the percentage of time spent in the open arms and the mean velocity in the open arms. Altered reactivity is calculated by combining the percentage of freezing and escaping scores when animals were exposed to a novel environment at D16. Altered arousal combines the scores for the maximum amplitude response ratio with the latency of maximum responses ratio during the airpuff startle session. Each individual variable score ranged from 0 to 3, and the combined score ranged from 0 to 6.

### 2.4 Sleep recordings

#### 2.4.1 Electrodes implantation

All mice (n = 47) were anesthetized with isoflurane (4% induction, 1–2% maintenance) and given buprenorphine (0.05 mg/kg, s.c.) and carprofen (4 mg/kg, s.c.) preoperatively. After shaving and cleaning, animals were placed in a stereotaxic frame. EEG electrodes (frontal: AP +2, ML +1.5; parietal: AP –2, ML –1.5; reference: AP –6, ML +2) were secured with screws, and EMG wires were inserted into neck muscles. Electrodes were connected to a head-mounted connector (MS363, PlasticOne) and fixed with Superbond® dental cement. Postoperatively, mice recovered and were monitored for 15 days.

#### 2.4.2 The recording system

Recordings were performed in a dedicated room, with animals housed individually in recording boxes throughout the procedure. Signals were amplified ( × 1000; model 3500, AM Systems), filtered (0.3–200 Hz), digitized at 1024 Hz (Micro1401 Power3, CED), and visualized with Spike2 (v7, CED).

#### 2.4.3 Recording Day

To minimize stress from handling, animals were habituated for one week to both the handling required for connecting them to the recording equipment and to their recording boxes. On day before recording, animals were manually restrained and briefly connected to the equipment. Recordings began at 10:00 a.m. (lights on) the day after. Two 24-h sleep recordings were obtained: one week before foot-shock (Baseline) and on day 14 (D14). On Day 14, mice were exposed to the FS grid for 5 min as a cue reminder to model PTSD-like intrusions and potential sleep disruption (Kobayashi et al., 2007; Philbert et al., 2011; Zhang et al., 2019). Sleep recordings began immediately afterward to maximize the likelihood of detecting stress-induced sleep alterations (Philbert et al., 2011).

#### 2.4.4 Polysomnographic analyses (baseline and D14)

Vigilance states (wakefulness, slow-wave sleep, paradoxical sleep) were scored from EEG/EMG recordings in 5-s epochs using a validated MATLAB algorithm (r2019, MathWorks®) (Claverie et al., 2023). States were identified by spectral features of frontal EEG and EMG: wakefulness (high, variable EMG; low-amplitude, fast EEG with 4–8 Hz activity during exploration), slow-wave sleep (low EMG; high-amplitude EEG with 1–4 Hz oscillations and 10–14 Hz spindles), and paradoxical sleep (very low EMG; low-amplitude EEG dominated by 5–9 Hz theta). Animals with poor quality EEG signals were excluded. Automated scoring was visually verified by an experimenter blind to the group. Vigilance states were analyzed over 24 h, quantifying percentage of time, episode number and duration, and sleep onset latency (first ≥25 s episode after recording start). At baseline, latency to the first sleep episode was measured from the time the lights were switched on. Fourteen days after stress exposure, latency was measured from the time animals were exposed to the cue reminder, which was placed in the recording box at lights-on.

#### 2.4.5 Radar plot of sleep scores (Fig. 5G)

The radar plots represent the median scores for sleep entry and sleep continuity during both SWS and REM sleep 14 days after stress exposure. The sleep entry score was calculated by summing the number of episodes and the latency of the first sleep episode. The sleep continuity score was calculated by summing the percentage of time spent and the mean duration of sleep episodes. Score thresholds (0, 1, 2, and 3) for all variables were defined based on the median, standard deviation, and the 5th, 25th, 75th, and 95th percentiles of the No-FS dataset. Each individual score ranged from 0 to 3, then the combined scores ranged from 0 to 6.

### 2.5 Cellular staining

#### 2.5.1 Brain pretreatment

90 min after full context exposure, mice were deeply anesthetized with 4% isoflurane, perfused with 1X PBS (no fixative), and brains were rapidly collected, fresh-frozen in −25 °C isopentane, then stored at −80 °C. Three cryostat sliced 20 μm adjacent sections were obtained from the bilateral amygdala (Bregma −1.34 to −1.58 mm) and periaqueductal gray (posterior PAG: −4.96 to −5.02 mm; anterior PAG: −4.48 to −4.36 mm).

#### 2.5.2 Immunostaining

Slices were post-fixated with 4% PFA at 4 °C for 1 h before proceeding to immunostaining. After saturation (Emerald® antibody diluent, cat. #936B-08, Sigma-Aldrich), an overnight cold (4 °C) incubation of primary antibodies was carried out: rabbit anti-c-Fos, cellular activation marker (1:1000; Abcam, cat. # ab190289), and mouse Ig2b-anti-PKCdelta, central lateral amygdala marker (1:1000; BD Biosciences, cat. #610398). The next day, a 2-h secondary incubation was performed: Donkey anti-rabbit IgG (Alexa Fluor™ 568, 1:2000; Thermo Fisher Scientific, cat. #A10042), and goat anti-mouse IgG2 (Alexa Fluor™ 488, 1:1000; Thermo Fisher Scientific, cat. #A2114).

#### 2.5.3 Staining analysis

Confocal images were acquired on an Olympus W1 spinning disk microscope using a 40 × /1.4 NA objective. Tile scans were stitched in Zen 3.0. c-Fos-positive nuclei were quantified with a custom FIJI workflow involving smoothing, local maxima detection, and watershed-based binary mask processing; DAPI and c-Fos masks were combined by logical AND. Parameters were optimized for PAG and AMG, and the custom script is available upon request. PKC-δ staining delineated CeL from CeM, while LA, BA, and PAG boundaries were defined using the Paxinos and Franklin (2019) atlas. For each animal and region, the median of three slides was calculated. Only animals with complete behavioral data, analyzable sleep recordings, and high-quality AMG and PAG staining were included.

#### 2.5.4 Radar plot of the cellular activation score (Fig. 6I)

Cellular activation scores reflect the number of c-Fos–positive cells quantified in each region of interest within the amygdala and periaqueductal gray (PAG). Score thresholds were defined based on the median, 25th percentile, and 75th percentile of the No-FS datasets. Scores from the basoanterior (BA) and lateral nuclei (LA) were combined to generate a basolateral amygdala (BLA=BA + LA) score, whereas scores from the central lateral (CeL) and central medial (CeM) nuclei were combined to generate a central amygdala (CeA=CeL + CeM) score. For the PAG, scores from the anterior and posterior subdivisions of the ventral PAG and from the dorsal PAG were combined to generate ventral and dorsal PAG scores, respectively. Each individual score can go from 0 to 3, then the combined scores from 0 to 6.

### 2.6 Statistical analysis

The threshold for statistical significance is p < 0.05. Non-parametric tests were used due to heterogeneous variances. Intragroup comparisons were performed with paired Wilcoxon or Friedman tests (£). Intergroup differences were systematically assessed across all four groups using Kruskal–Wallis ANOVA, followed by post hoc comparisons (∗). Comparisons of group means to a theoretical value are indicated by #. For each intragroup comparison, the median of each data group is shown in the figures and indicated by a black line. Individual data points are also plotted. For behavioral data with larger animals per group, error bars represent the interquartile range (IQR). For immunostaining data with smaller sample sizes (n = 4 per group), only individual data points and the group median are shown. For sleep analysis, individual data points are shown together with IQR. Significant Spearman correlations between behavioral, cellular, and sleep measures are marked with $; tendencies (0.05 < p < 0.07) are reported with the exact p-value. P-values below 0.0001 were shown as ∗∗∗/£££ in figures and reported as *p* < 0.0001 in the text for clarity. Analyses were conducted in Statistica®, and Figures were generated in GraphPad Prism® v8.0.2.

## 3 Results

### 3.1 A 2-step behavioral phenotype analysis highlighted three phenotypes in foot-shocked (FS) mice

To assess PTSD-like behavioral alterations following foot-shock exposure, we calculated a composite score by summing eight behavioral scores from four different tests designed to reflect DSM-V–like criteria (Fig. 2A, see Materials and Methods for details) allowing classification of animals as resilient or susceptible. For each test, a four-point score was assigned to two selected variables per animal (Fig. 2A). Among 31 foot-shocked animals, 20 were classified as susceptible (64.5%), and 11 as FS-Resilient (FS-Res, 35.5%) (Fig. 2F).

**Fig. 3:**
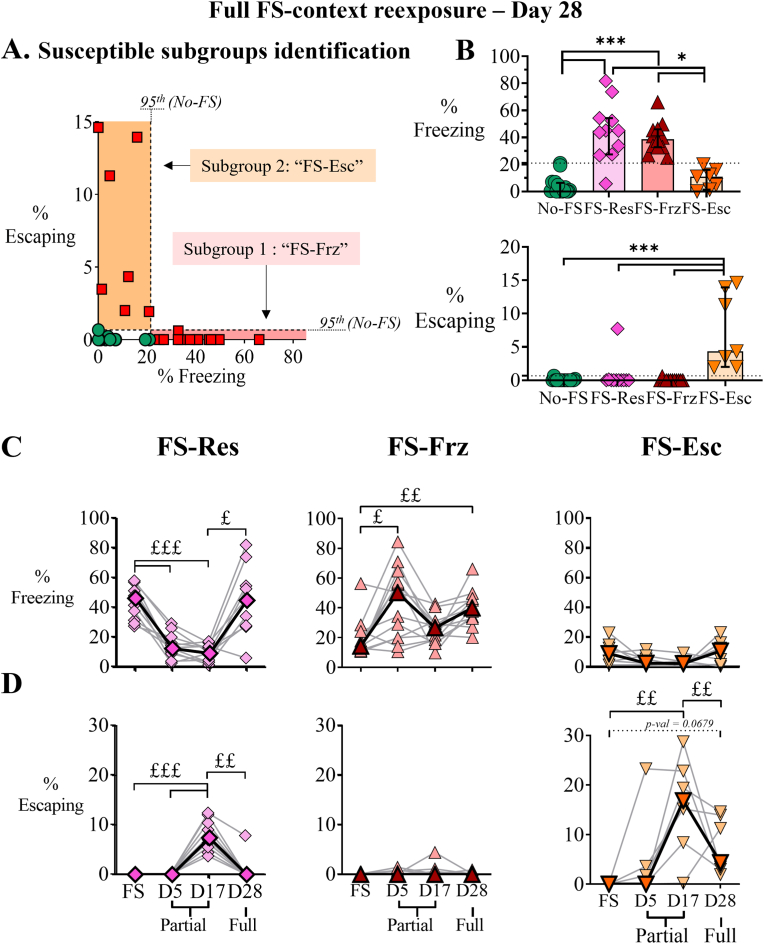
Second step of profiling behavioral analysis and characterization of two profiles among susceptible phenotypes through their defensive behavior. **A.** Behavioral phenotypes analysis of FS susceptible mice (□) according to their defensive behavior the day of the full context exposure (day 28). A FS animal was considered as “escaper” (FS-Esc) when presented with at least one escape attempt. The thresholds were decided according to 95th percentiles of the control group data set (dotted lines). **B.** The defensive behaviors (top, freezing and bottom, escaping) on day 28 were then analyzed with the two new subgroups. **C-D.** Percentage of freezing (C) and escaping (D) through all the procedure, the day of the foot-shocks exposure (FS), during the partial (day 5 and 17) and full (day 28) context exposure. Kruskal-Wallis post hoc analysis (∗) and Friedman post hoc analysis (£), ∗/£ p-val <0.05; ££, p-val <0.01, ∗∗∗/£££, p-val <0.001. Each light-colored symbol represents an animal, and the dark colored the median of the cluster. ໐ No-FS, no foot-shocks exposed mice (control) n = 16; ◇ FS-Res, foot-shocked resilient mice, n = 11; △ FS-Frz, foot-shocked susceptible freezing mice, n = 13 and ▽ FS-Esc, foot-shocked susceptible escaping mice, n = 7.

Afterward, FS susceptible animals were discriminated according to two observed defensive behaviors to the full foot-shock-associated context re-exposure on day 28 (Fig. 3A), leading to the characterization of two susceptible subgroups: animals with either a high rate of freezing, named Freezers (FS-Frz), or animals that had a low rate of freezing but tried to escape at least once during the session, named Escapers (FS-Esc). These results were statistically validated by Kruskal-Wallis ANOVA tests (Fig. 3B-top, H(3) = 32.59, p-val <0.0001; Fig. 3B-bottom, H(3) = 27.82, p-val <0.0001). Indeed, FS-Frz presented a higher percentage of freezing than both No-FS (Fig.3B, p-val <0.0001) and FS-Esc (Fig. 3B, p-val = 0.0265), while FS-Esc showed significantly more escaping percentage than No-FS (Fig. 3C, p-val <0.001), FS-Frz (Fig. 3B, p-val <0.0001) and FS-Res (Fig. 3B, p-val = 0.0002). Furthermore, FS-Res animals also presented a high percentage of freezing compared to both No-FS (Fig. 3B, p-val <0.0001) and FS-Esc (Fig. 3B, p-val = 0.0171), while showing no escaping behavior (Fig. 3B). The two defensive behaviors were then retrospectively investigated to examine if animals preserved the same behavior throughout the entire experimental procedure. The percentages of freezing (Fig. 3C) and escaping (Fig. 3D) were then assessed during partial context exposures occurring on days 5 (D5) and 17 (D17; partial context exposures), but also on the day of foot-shock exposure (FS). We observed that the percentage of freezing of FS-Frz was increased on D5 and D28 compared to FS day (Fig. 3C; Friedman analysis: X^2^ = 13.89, p-val = 0.0031; FS day vs. D5: p-val = 0.0234; vs. D28 p-val = 0.0050), without showing significant escaping behavior during all the procedure time (Fig. 3D). On the contrary, FS-Esc presented an increased percentage of escaping on days 17 compared to FS days and D28 (Fig. 3D; X^2^ = 15.36, p-val = 0.0015; D17 days vs. FS: p-val = 0.0026 and vs. D28: p-val = 0.0026), while presenting low percentages of freezing during from FS days to D28 (Fig. 3C). A tendency was observed when the percentage of escaping on D28 and FS days were compared (p-val = 0.0679). Regarding FS-Res, the percentage of freezing was decreased on days 5 and 17 compared to the day of FS exposure (Fig. 3C; X^2^ = 24.27, p-val <0.0001; FS days vs. D5: p-val = 0.009 and vs. D17: p-val = 0.0001), with an increase between D17 and D28 (p-val 0.03), whereas the percentage of escaping was increased on day 17 compared to FS days and D5 (Fig. 3D; X^2^ = 30.75, D17 vs. FS: p-val = 0.0009 and vs. D5: p-val = 0.0009), following by a decrease on D28 (p-val = 0.0057. FS-Frz and FS-Esc mice consistently, for the most part, displayed their respective defensive behaviors from the day of intensive stress through the end of the procedure. These stable responses were therefore defined as the characteristic behavioral phenotypes of each group. Based on this second step analysis, FS mice were classified into three distinct phenotypes: 11 as FS-Resilient (FS-Res, 35.5%), 13 as FS-Freezer (FS-Frz, 42%), and 7 as FS-Escaper (FS-Esc, 22.5%).

**Fig. 4:**
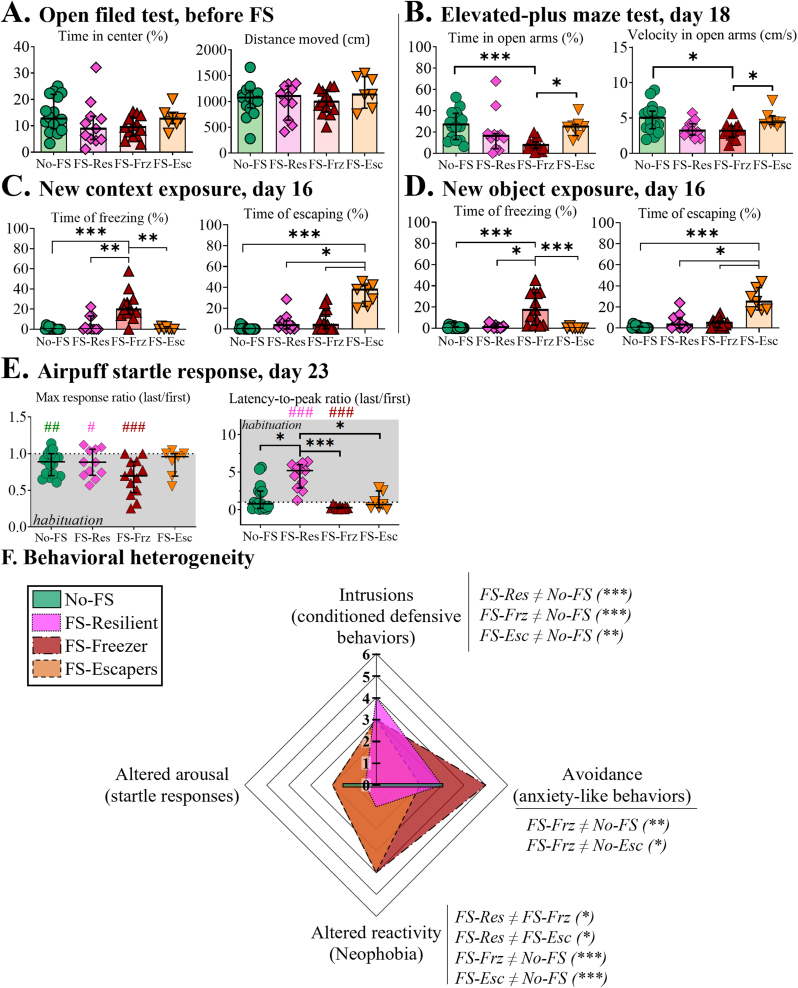
Differential behavioral alterations for each phenotype. To statistically confirm the heterogeneity of behavioral alterations of the three highlighted phenotypes, a Kruskal-Wallis-test on each variable was performed: **A-E**. Anxiety-like responses before (Open-field, A) and after (EPM, B) foot-shock exposure (FS), new context (C) and new object (D) and startle responses (E). For each group, the median value (bar plot or a line) is represented. Each symbol stands for an animal: ໐ No-FS, no foot-shock exposed mice (control) n = 16; ◇ FS-Res, foot-shocked resilient mice, n = 11; △ FS-Frz, susceptible freezing mice, n = 13 and ▽ FS-Esc, susceptible escaping mice, n = 7. Kruskall-Wallis post-hoc analysis (∗) and comparisons to a theoretical median of 1 (#) post-hoc analysis: ∗/#, p-val< 0.05; ∗∗/##, p-val<0.01 and ∗∗∗/###, p-val<0.001. **F.** Radar chart illustrating the median scores obtained from each behavioral test. Tests evaluated distinct PTSD-like behavioral dimensions in rodents: intrusion (cue reminder – grid exposure), avoidance (EPM), altered reactivity (new context/object), and altered arousal (startle response). Post hoc comparisons following the Kruskal–Wallis test are reported below each parameter: a ≠ b indicates a significant difference between groups a and b; ∗, p < 0.05; ∗∗, p < 0.01; ∗, p < 0.001; ns, non-significant. The radar chart analysis is explained in more details in the Material and methods section.

**Supplementary Fig. 1:**
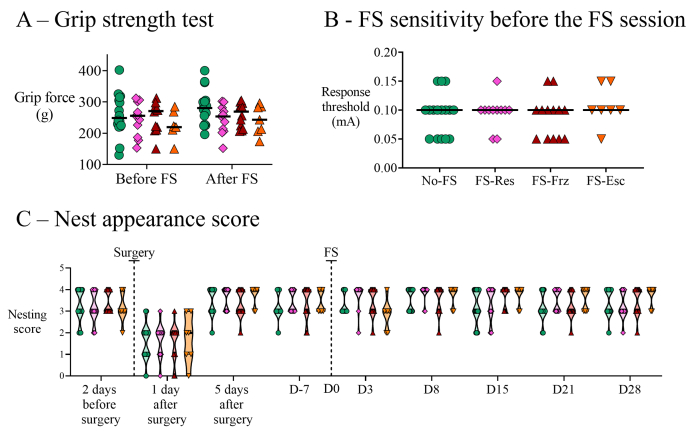
Behavioral responses before and after the FS exposure. To assess a potential marker of stress vulnerability before the intense stress exposure (foot-shocks, FS), animals spent three additional tests: a grip test, to assess the paws muscle strength using Bioseb system (France, BIO-GS4) (A); a FS sensitivity test (B; one week before the intensive FS exposure and the assessment of the nest appearance (C) (Nollet, 2021). The solid lines indicate the median, and each symbol represents an animal: ໐ No-FS, no foot-shocks exposed mice (control), n = 16; ◇ FS-Res, foot-shocked resilient mice, n = 11; △FS-Frz, foot-shocked susceptible freezing mice, n = 13 and ▽ FS-Esc, foot-shocked susceptible escaping mice, n = 7.

Finally, the potential differential expression of behavioral alterations was assessed for the three phenotypes (Fig. 4). First, the possibility of a pre-existing marker of stress resilience/susceptibility was examined in an open field experiment performed 7 days prior to FS exposure (Fig. 4A). Both ANOVA of the percentage of time spent in the center of the open field and the total distance moved in the apparatus for 5 min did not reveal significant results (H(4) = 5.721, ns and H(4) = 2.069). No difference in foot-shock sensitivity, muscular strength, nor nest building behavior was detected before and after foot-shocks. (Sup. Fig. 1).

Then, the behaviors of the newly defined phenotypes were assessed in the scored post-FS tests (Fig. 4) to validate the observed behavioral heterogeneity. A significant group effect was observed by ANOVA in the EPM on day 18 for both the percentage of time spent in the open arms and the mean velocity in open arms (Fig. 4B, Kruskal-Wallis non-parametric tests, H(3) = 17.67, p-val = 0.0005 and H(3) = 12.52, p-val = 0.0058, respectively). The post hoc analysis revealed that FS-Frz mice spent less time in the open arms of the EPM than both the control group and FS-Esc mice (p-val = 0.0005 and p-val = 0.0133, respectively). Furthermore, the mean velocity of FS-Frz in the open arms was inferior to both the control and FS-Esc mice (p-val = 0.0223 and p-val = 0.0436, respectively). For the new context and the new object (Fig. 4C and D), the Kruskal Wallis of the two defensives behaviors, percentage of freezing and escaping, revealed significant results for both the new context (Fig. 4C; freezing: H(3) = 23.24, p-val <0.0001 and escaping: H(3) = 22.28, p-val <0.0001) and the new object (Fig. 4D; freezing, H(3) = 27.37, p-val <0.0001 and escaping, H(3) = 23.10, p-val <0.0001). The post-hoc analyses revealed that the FS-Frz mice spent more time freezing compared to No-FS but also FS-Res and FS-Esc mice when exposed to both a new context (p-val <0.0001, p-val = 0.0072 and p-val = 0.0021, respectively) and a new object (p-val <0.0001, p-val = 0.0291 and p-val <0.0001, respectively). On the contrary, the FS-Esc animals spent more time trying to escape the arena compared to No-FS, FS-Res and FS-Frz when exposed to contextual novelty (p-val <0.0001, p-val = 0.0317 and p-val <0.0154, respectively) or a new object (p-val <0.0001, p-val = 0.0109 and p-val <0.0179, respectively).

Eventually, the Kruskal-Wallis ANOVA of the startle response revealed no significant differences for the ratio of the maximum responses (Fig. 4E; H(3) = 3.080, ns). However, intra-phenotype analyses revealed that this ratio for FS-Res, FS-Frz and the control groups was significantly inferior to 1, signifying a decrease in the startle responses during the session (Fig. 4E left, p-val = 0.0474, p-val = 0.001 and p-val = 0.0067, respectively), in contrast to FS-Esc mice, which did not express any such behavioral habituation to the airpuff. A significant group effect for the latency-to-peak ratio was also found (Fig. 4E right, H(3) = 21.17, p < 0.0001). Specifically, the latency ratio values of FS-Res mice were higher than in the FS-Frz (p-val <0.0001), FS-Esc (p-val = 0.0292) and No-FS mice (p-val = 0.0112). Furthermore, the median of the latency ratio of FS-Res was higher to 1 (p-val = 0.0010), while the FS-Frz value was inferior (p-val = 0.0010), meaning respectively an increase and a decrease in response latency to the airpuff during the session. To provide an overview of the differential behavioral expression, the scores for each test were represented with a radar chart (see Material and methods for detailed analysis, Fig. 4F). Both susceptible and resilient mice expressed a high level of conditioned defensive behaviors score (intrusion-like symptom) when exposed to FS-related stimuli (Kruskal-Wallis: H(3) = 32.99; No-FS vs. FS-Res: p-val <0.0001; vs. FS-Frz: p-val <0.0001 and vs. Fs-Esc, p-val = 0.0019). The susceptible freezer phenotype showed a significant anxiety-like behavior score in the elevated-plus maze (avoidance-like symptom, H(3) = 13.32; p-val = 0.004) compared to both susceptible escaper animals (p-val = 0.028) and control group (p-val = 0.0087). Furthermore, even if presenting different defensive responses, both FS susceptible FS-Frz and FS-Esc showed a higher score of neophobia (altered reactivity like symptom, H(3) = 31.55, p-val <0.0001): both FS-Frz and FS-Esc median scores were higher than FS-Res (p-val = 0.0425 and p-val = 0.0491, respectively) and No-FS (p-val <0.0001 and p-val = 0.00014, respectively). The startle response score (altered arousal-like symptom) score tended to be different between groups (H(3) = 7.64, p-val = 0.0542).

### 3.2 The classification of FS mice revealed specific sleep patterns in resilient mice

**Fig. 5:**
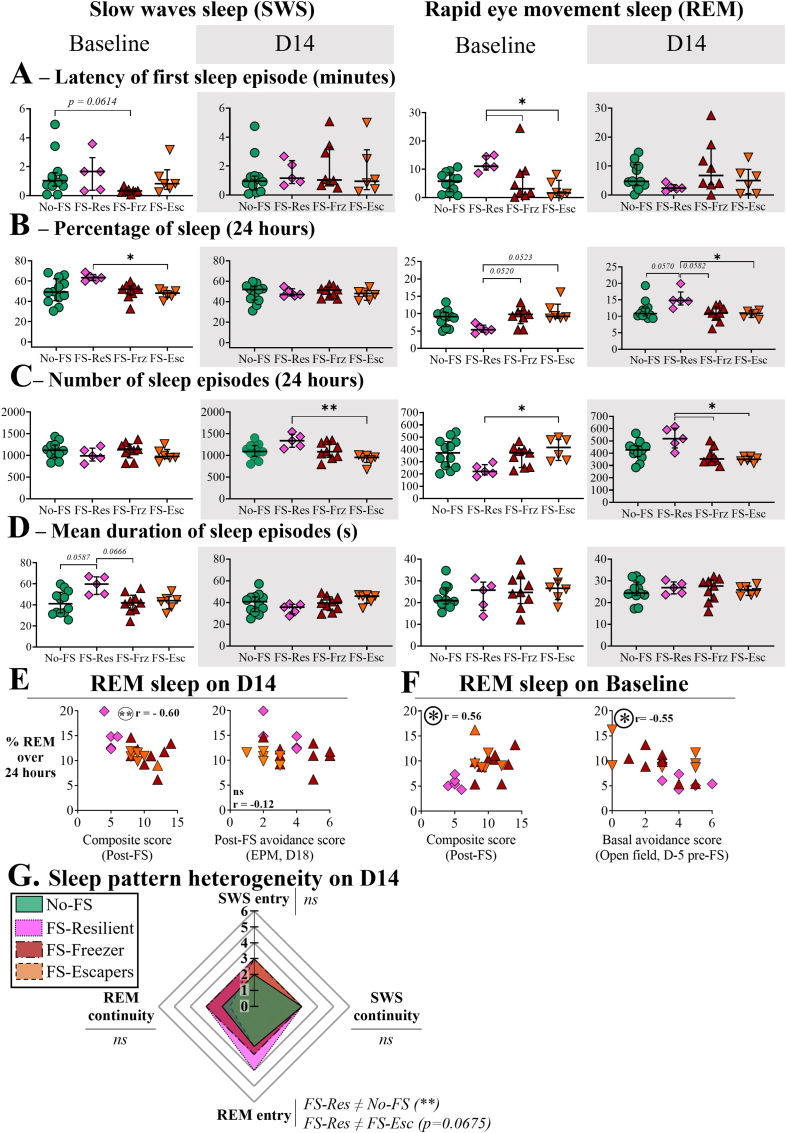
Sleep analyses prior and 14 days after the FS exposure. **A.** Latency of the first slow wave sleep (SWS, left) and rapid eye movement sleep (REM, right) episode in minutes. **B-D.** Percentage of time spent (B), number (C) and mean duration in seconds (D) of SWS and REM over 24 h. **E-F.** Spearman correlations between the REM sleep 14 days after (D14) and before (baseline) the FS exposure with the composite score and pre/post-FS anxiety-like behavior scores (obtained with open-field, D-5 and elevated-plus maze D18 tests respectively). Intergroup Kruskal-Wallis post-hoc analysis: ∗, p-val<0,05; ∗∗: p-val<0,01. Spearman correlations: r, correlation coefficient; circled ∗, p < 0.05; circled ∗∗, p < 0.01. Each symbol represents an animal, ໐ No-FS, no foot-shocks exposed mice (control), n = 12. ◇ FS-Res foot-shocked resilient mice, n = 5; △ FS-Frz, foot-shocked susceptible freezing mice, n = 9 and ▽ FS-Esc, foot-shocked susceptible escaping mice, n = 6. The light tones represent the baseline data and the dark tones the data for D14. **G.** Radar chart illustrates the median scores obtained for sleep entry (sum of latency and number of sleep episode scores) and sleep continuity (sum of percentage of sleep and mean duration of sleep episodes scores) in both REM and SWS. Post hoc comparisons following the Kruskal–Wallis test are reported below each parameter: a ≠ b indicates a significant difference between groups a and b; ∗, p < 0.05; ∗∗, p < 0.01 and ns, non-significant. The radar chart analysis is explained in more details in the Material and methods section.

The sleep patterns of each mouse phenotype were assessed before (baseline, white) and 14 days after (D14, gray) foot-shock exposure (Fig. 5A–D). Two main sleep stages were analyzed over 24 h: slow-wave sleep (SWS, two leftmost columns) and rapid eye movement sleep (REM, two rightmost columns) and for the four experimental groups. The analysis of the SWS latencies revealed only that FS-Frz animals tend to fall into SWS earlier than No-FS during baseline (Fig. 5A; H(3) = 8.638, p-val = 0.0345; No-FS vs FS-Frz, p-val = 0.0614). The analysis of the REM latencies during baseline revealed also significant differences between groups (H(3) = 10.73, p-val = 0.0133). Precisely, FS-Res animals REM latency was higher than both FS-Frz (p-val = 0.0433) and FS-Esc (p-val = 0.0116).

Group comparisons of sleep parameters were performed at baseline and 14 days after foot-shock exposure using Kruskal–Wallis tests. First, regarding the percentage of time spent in SWS (Fig. 5B), Kruskal-Wallis analysis highlighted a significant group effect on baseline (H(3) = 9.181, p-val = 0.0270), but not on D14 (H = 1.053, ns). Specifically, FS-Res spent more time SWS than FS-Esc (p-val = 0.02696). Regarding the number of SWS episodes (Fig. 5C), the intergroup analysis revealed no difference before but did so after FS exposure (H = 2.317, ns and H = 11.11, p-val = 0.0112, respectively). Indeed, FS-Res has more SWS episodes than FS-Esc over 24 h 14 days post-stress (p-val = 0.0053). Furthermore, the mean duration of SWS episodes were also different before FS exposure (Fig. 5D, H = 8.033, p-val = 0.0453) but not 14 days post-stress (H = 5.155, ns). Specifically, the average SWS episode duration in the FS-Res group was slightly longer than No-FS (p-val = 0.0587) and FS-Frz (p-val = 0.0666) before exposure.

Regarding the REM sleep (right figures), the analysis of the percentage of time spent in REM unveiled significant differences before (H = 8.686, p-val = 0.0338) and after FS exposure (H = 9.715, p-val = 0.028). Specifically, before FS exposure, FS-Res animals tended to spend less time in REM sleep than the two susceptible groups, FS-Frz (p-val = 0.0520) and FS-Esc (p-val = 0.0523). Interestingly, FS-Res animals spent more time in REM sleep than FS-Esc animals (p = 0.0261) and showed a trend toward increased REM sleep compared with FS-Frz animals (p = 0.0582) on day 14. Furthermore, the non-parametric ANOVA of the number of REM sleep episodes revealed differences before (H = 8.570, p-val = 0.0356) and 14 days after FS exposure (H = 11.66, p-val = 0.0086). Precisely, the number of REM episodes of FS-Res animals were lower than FS-Esc (p-val = 0.0307) before stress exposure but were higher than both FS-Esc (p-val = 0.0119) and FS-Frz (p-val = 0.0292) 14 days after the foot-shocks. Finally, non-parametric ANOVA of the mean duration of REM episodes did not reveal any group effect before or after FS exposure (Fig. 5D; H = 1.895, ns and H = 0.679, ns respectively).

Further sleep analysis was performed with correlations between all sleep parameters and behavioral data, revealing significant results only with REM (Fig. 5E and F). The percentage spent in REM on day 14 was negatively correlated with the composite score (Fig. 5E, Spearman coefficient r = -0.6002, p-val = 0.0051; Fig. 5E, left), but not to the post-FS avoidance-like score (EPM score). On the contrary, the percentage of REM on baseline was positively correlated with the composite score (Fig. 5F, r = 0.5578, p-val = 0.0106) and negatively correlated with the baseline avoidance score (open field, D-7) before the foot-shock exposure (r = −0.55, p-val = 0.0144; Fig. 5F, right).

Using the same scoring method as for the behavioral data, we performed an ANOVA on the median scores for sleep entry (sum of number of sleep episodes and latency scores) and sleep continuity (sum of percentage of sleep and mean duration scores) for both SWS and REM sleep 14 days post-FS (Fig. 5G, see Materials and Methods for details). The analysis revealed a median score significantly different for the REM entry (H(3) = 10.70, p-val = 0.0135). Precisely, FS-Res REM entry score was higher than score of No-FS (p-val = 0.0084), and presented a trend toward higher score than FS-Esc score (p-val = 0.0675). The Kruskal–Wallis test revealed a near-significant effect of group on REM continuity and SWS entry scores (REM continuity: p-val = 0.0561; SWS entry: p-val = 0.0686); however, post-hoc pairwise comparisons did not reveal significant nor trend differences between groups.

### 3.3 The classification of FS mice revealed different brain cellular activities

**Fig. 6:**
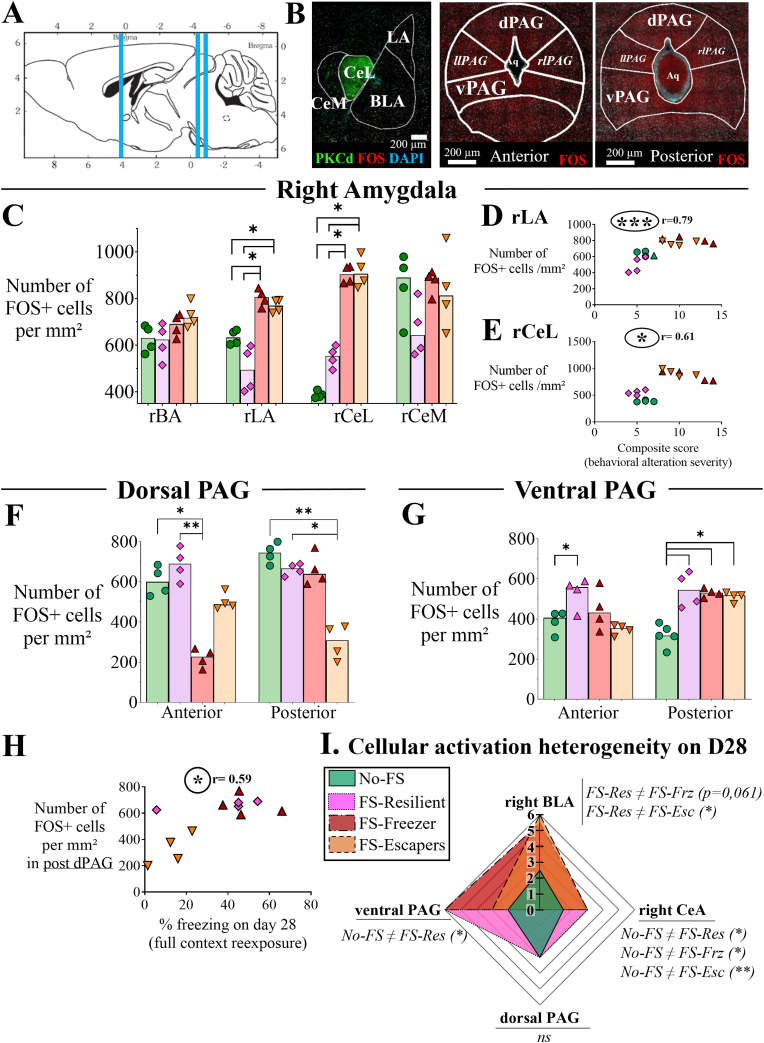
Differential cellular activations of amygdala and periaqueductal gray matter after full FS-associated context re-exposure on day 28. **A.** Sagittal view of the mice brain, according to the Allen atlas. Representation of the right amygdala (rAMG) and the anterior and posterior periaqueductal gray matter (PAG) zones of interest. **B.** Brain slices focusing on the four main right (r) AMG nuclei: lateral (rLA), basolateral (rBLA), central lateral (rCeL) and central medial (rCeM) and the 4 columns of the PAG: dorsal, dPAG, ventral, vPAG, and left/right lateral, llPAG/rlPAG) in anterior (Bregma −4.48 mm to −4.36 mm) and posterior (Bregma −4.96 mm to −5.02 mm) parts. Fluorescent immunohistochemistry of PKCd (green) delimits the CeL borders, c-Fos (red) as recently activated neuron marker and DAPI (blue) as nuclear maker. Scale bars: 200 μm. **C.** Number of c-Fos positive (c-Fos^+^) cells per mm^2^ in the four right AMG nuclei. **D-E.** Spearman correlations between the number of c-Fos ^+^ cells per mm^2^ in the rLA (D) or rCeL (E) and the behavioral composite score. **F-G.** Number of c-Fos ^+^ cells per mm^2^ in the dorsal PAG (F) and the ventral PAG (G). **H.** Spearman correlation between the posterior part of the dPAG and the percentage freezing on day 28 during the full context re-exposure. Kruskal-Wallis post hoc analysis: ∗, p-val<0,05; and ∗∗, p-val<0,01. Spearman correlations: r, correlation coefficient; circled ∗, p < 0.05; circled ∗∗, p < 0.01 and r stands for the Spearman statistical value. Each symbol indicates the median of three slice counts per animal, and each bar represents the median of the group. ໐ No-FS, no foot-shocks exposed mice (control). ◇ FS-Res, resilient mice, ▽ FS-Frz, susceptible freezing mice and ▽FS-Esc, susceptible escaping mice. n = 4 for each group. **I.** Radar chart illustrating the median scores obtained from the cellular activation on day 28 in the right basolateral (BLA; sum of BA and LA activation scores), right central amygdala (CeA; sum of CeL and CeM activation scores) dorsal PAG and ventral PAG. Post hoc comparisons following the Kruskal–Wallis test are reported below each parameter: a ≠ b indicates a significant difference between groups a and b; ∗, p < 0.05; and ns, non-significant. The radar chart analysis is explained in more details in the Material and methods section.

First, AMG activity was resolved into 4 anatomo-functional nuclei (BA, LA, CeL and CeM; Fig. 6B). We distinguished between the left and right amygdala. No significant differences were observed between groups in the left (l) amygdala nuclei (H(lBA) = 0.2035, H(lLA) = 5.00, H(lCeL) = 0.9804 and H(lCeM) = 0.6405, data not shown). In contrast, ANOVA revealed a significant group effect in right LA (Fig. 6C, rLA, H(3) = 8.914, p-val = 0.031) and right CeL (Fig. 6C, rCel, H = 11.30, p-val = 0.010). Specifically, the number of c-Fos-positive (c-Fos+) cells in the rLA was higher for both FS-Frz and FS-Esc in comparison to FS-Res (p-val = 0.022, p-val = 0.034, respectively) and to No-FS (p-val = 0.046, p-val = 0.045, respectively). Similarly, the number of rCeL c-Fos + cells was higher in FS-Frz and FS-Esc than FS-Res (p-val = 0.048, p-val = 0.038, respectively) and No-FS (p-val = 0.041, p-val = 0.018, respectively). Interestingly, a positive correlation was found between the density of c-Fos cells in the rLA and the rCeL nuclei and the composite score (Fig. 6D and E, Spearman coefficient r = 0.79, p-val = 0.0004 and r = 0.61, p-val = 0.014, respectively). In contrast, no correlation was observed between AMG c-Fos expression and freezing on day 28 (0.006 < r < 0.07 for the four rAMG nuclei; ns: data not shown).

The dorsal and ventral PAG cellular sub-region activities (dPAG and vPAG) were analyzed between groups in two regions, referred to as the anterior and posterior parts (Fig. 6B). First, differential cellular activation variations were found between groups in both the anterior and posterior parts of the dPAG (Fig. 6F; H = 7.49, p-val = 0.049 and H = 11.54, p-val = 0.009, respectively). In the anterior part, the intergroup analysis revealed that cellular activation in the FS-Frz phenotype was lower compared to No-FS (p-val = 0.048) and FS-Res (p-val = 0.0036). In the posterior part of the dPAG (Fig. 6F), FS-Esc cellular activation was lower compared to No-FS (p-val = 0.0041) and FS-Res (p-val = 0.048).

ANOVA revealed a significant group effect in both the anterior and posterior parts of the vPAG (Fig. 6G; H = 13.39, p-val = 0.0039 and H = 10.41, p-val = 0.0154, respectively). Only FS-Res mice presented a higher positive cell rate than the control group in the anterior vPAG (Fig. 6E; p-val = 0.0451). In the posterior vPAG (Fig. 6G), the 3 FS phenotypes presented more c-Fos + cells than No-FS mice (FS-Res: p-val = 0.049, FS-Frz p-val = 0.030 and FS-Esc: p-val = 0.049).

**Supplementary Fig. 3:**
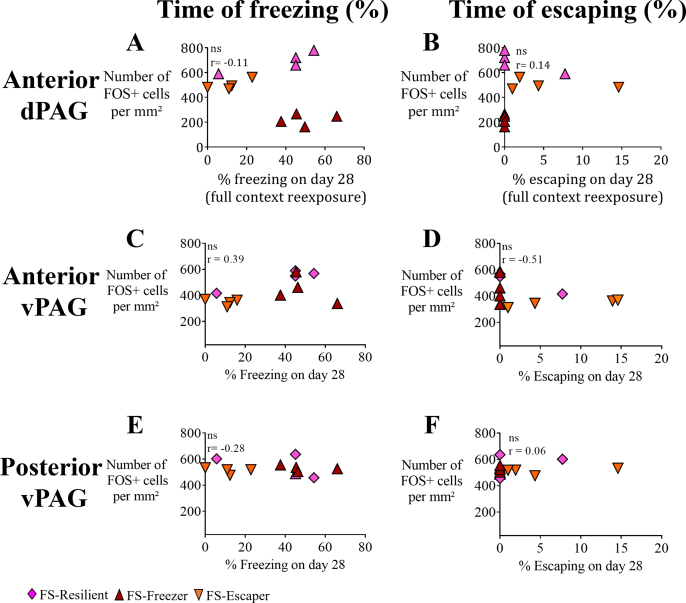
The ventral PAG cellular activations are not correlated to the defensive responses the day of the full context re-exposure. Spearman correlations between caudal anterior (left) or posterior (right) vPAG cellular activities with the percentage of time spent freezing (A) or the percentage of time spent escaping (B). r: Spearman correlation coefficient. ns for non-significant p value.

We then assessed the correlation of cellular activity and defensive behaviors on day 28 (freezing and escaping) (Fig. 6H). No significant correlation was found for the anterior vPAG correlation analysis regarding percentage of time spent freezing, or in the anterior part of the dPAG (Sup. Fig. 3). In contrast, in the posterior part of the dPAG (Fig. 6H), the Spearman analysis revealed a significant positive correlation between the number of c-Fos + cells and the percentage of time spent freezing on day 28 (r = 0.5874, p-val = 0.0489).

Following the same logic of the behavioral scoring method, the median cell activation scores in right amygdala subnuclei (basolateral and central) and PAG subdivisions (ventral and dorsal) were compared (Fig. 6I; see Materials and Methods for details). In the amygdala, both activation scores differed significantly across groups (BLA: H(3) = 12.34, p = 0.0003; CeA: H(3) = 12.94, p = 0.0022). Specifically, the BLA activation score of resilient animals was lower than that of FS-Esc animals (p = 0.0226) and showed a trend toward higher activation compared to FS-Frz animals (p = 0.0612). Furthermore, the CeA scores of all three FS groups were higher than the control group (FS-Res and FS-Frz: p = 0.0422; FS-Esc: p = 0.0068). Significant differences were also observed in ventral PAG activation scores (H(3) = 7.965, p = 0.0314), with FS-Res animals showing higher scores than No-FS animals (p = 0.0355).

## 4 Discussion

Our analysis of foot-shocked (FS) animals identified three phenotypes with distinct coping strategies, sleep profiles, and c-Fos activation patterns. Resilient mice displayed cue-specific defensive behaviors, increased REM sleep, and selective ventral PAG activation. Susceptible freezer mice generalized defensive responses, showed anxiety-like behavior, reduced SWS latency at baseline, and exhibited hyperactivation of the amygdala with decreased dorsal PAG recruitment. Susceptible escaper mice also generalized defensive behaviors but showed no sleep alterations, with strong amygdala activation and reduced dorsal PAG recruitment. These results provide a clear multi-level characterization of the three phenotypes.

Working with preclinical models of human psychiatric disorders presents substantial challenges, as translational validity remains a central concern across experimental paradigms (Borghans, 2015; Richter-Levin et al., 2019). We acknowledge that the use of electrical foot-shock does not represent the most ethologically relevant model of PTSD in rodents. However, the high degree of controllability and reproducibility it provides, particularly regarding the intensity and duration of stress exposure—as well as the capacity to dissociate contextual and cue-related reminders, were critical methodological advantages for the present study. Furthermore, this paradigm is known to both induce long-lasting behavioral alterations and sleep alterations that mimic PTSD-like symptoms as described in the DSM-5 (Pawlyk et al., 2008; Verbitsky et al., 2020) which are essential for establishing consistent behavioral phenotyping. This controlled framework allowed us to precisely examine how individual coping strategies, and their associated neural and sleep alterations, emerge following a standardized stress experience. Given the strong influence of the estrous cycle on stress responses, we focused on males in this initial study to avoid additional complexity, while recognizing the importance of including females in future work. The heterogeneity of PTSD-like behavioral alterations among stress-exposed animals was analyzed using individual behavioral profiling, which is crucial for understanding the neural basis of stress vulnerability and resilience (Ardi et al., 2016; Cohen et al., 2004; Richter-Levin et al., 2019). However, the variability in behavioral stress responses remains insufficiently explored in relation to biological and sleep correlates (see, however, Dopfel et al., 2019; Ritov et al., 2016; Wellman et al., 2016). Our profiling approach was motivated by numerous reports from various research groups highlighting heterogeneity in behavioral outcomes, particularly following stress exposure. In the present study, this heterogeneity was clearly expressed: escaper mice displayed frequent escape attempts, freezer mice showed pronounced freezing behavior, whereas resilient mice behaved similarly to non-stressed controls in the novel context test. Consequently, stressed animals could not be analyzed as a homogeneous group, and subgroup analyses were essential to reveal distinct neural and sleep activity patterns.

Our behavioral scoring system captures individual differences, reducing reliance on group averages and allowing identification of subpopulations that may show stress susceptibility in some assays but not others. However, converting complex behaviors into numerical scores can obscure nuances, such as inter-individual variability in escape responses. To address this, we used a 4-point scoring system and included continuous measures where possible. While using standard percentiles, we present individual data points to illustrate variability. While a one-step method is valid, we deliberately used a two-step approach to address distinct aims: first, to determine whether resilient and susceptible animals could be distinguished using no foot-shock-related tests, and second, to characterize susceptibility through trauma-specific defensive responses. This sequential strategy allowed us to separate general stress reactivity from foot-shock–specific behaviors. All FS animals exhibited freezing behavior on the day of foot-shock exposure, indicating successful fear induction. Although no major behavioral differences were observed between resilient and susceptible animals before or on the day of FS exposure (Sup. Fig. 1, Fig. 2), FS-resilient animals displayed significantly higher freezing levels during foot-shock delivery. This heightened acute defensive response likely reflects a more robust, context-appropriate reaction rather than differences in long-term behavioral outcomes. Consistent with previous studies distinguishing resilient and susceptible phenotypes (Colucci et al., 2020; Deslauriers et al., 2019; Jeong et al., 2020; Philbert et al., 2011; Siegmund and Wotjak, 2007; Sillivan et al., 2017; Torrisi et al., 2021; Verbitsky et al., 2020), classification in the present study relied on defensive responses and their specificity to threat-associated cues. Notably, resilient mice exhibited strong defensive behaviors during foot-shock exposure and upon re-exposure to FS-associated cues, without generalizing these responses to novel contexts or cues.

The second step of our profiling analysis was used to assign a specific behavioral response in FS susceptible animals that could be divided into two groups according to the nature of their defensive behavior: passive response for freezers, and an active response for escapers. Although personality traits exist in animals (Forkosh et al., 2019; Gosling, 2001; Mehta and Gosling, 2008), it seemed more appropriate, in our study, to refer to a behavioral phenotype, as we only looked at socially isolated animals. Our phenotype classification (freezers and escapers) is less complex and only considers one parameter (coping mechanism) that is constant throughout the entire procedure. Interestingly, the retrospective study of behavioral expressions on the conditioned defensive behaviors highlighted differential expressions between freezers, presenting an anxiety-like phenotype, and escapers presenting a hypervigilance phenotype after the foot-shock delivery. Escaping behavior of resilient mice on Day 17 suggested a sensitized response induced by high intensity foot-shocks (Kamprath and Wotjak, 2004) and probably accentuated by new exploration tests on Day 16. Interestingly, the escaping behavior was not observed for the full context reminder test suggesting that the high level of defensive behavior observed for the resilient mice on Day 28 might mainly depend on associative memory but not from a non-associative sensitized process.

Since numerous studies have shown that intensive stress alters sleep patterns in rodents, we wanted to explore further if foot-shocks differently impact the sleep of the three phenotypes. Fear sensitization has been suggested to influence sleep regulation. In a previous control experiment from our lab, we recorded sleep on Days 0, 7, 14, and 21 to determine whether sleep alterations preceded behavioral deficits in susceptible animals (data not shown). Sleep patterns in susceptible animals remained stable throughout this period, whereas resilient animals exhibited an increase in REM sleep from Day 0 until Day 21. These results indicate that the lack of differential sleep patterns observed in susceptible mice (FS-Frz and FS-Esc) in the present study is unlikely to be due to the timing of the Day 14 recording.

Foot-shock increased the REM sleep time in resilient animals, but it had no effect whatsoever on susceptible animals. Therefore, the behavioral phenotype analysis presented was crucial to reveal the subtle, delayed effect of stress (14 days later) on sleep in Swiss mice. Notably, sleep was previously shown to only be reorganized in C57Bl/6J resilient mice 12 days after social defeat (Bush et al., 2022). Similarly, REM sleep was significantly reduced in susceptible Wistar rats 13 days after the foot-shock procedure (Wellman et al., 2016) and in susceptible C57Bl/6J mice 5 days after social defeat (Henderson et al., 2017). REM sleep plays an important role in emotional regulation (Vandekerckhove and Wang, 2018). To model PTSD-like intrusions and their impact on sleep, mice were exposed to a 5-min FS cue reminder on Day 14, immediately prior to recording. While we recognized that this cue may enhance stress effects on sleep, susceptible animals did not show altered sleep patterns, whereas resilient animals did exhibit increased REM sleep, highlighting a translationally relevant distinction between phenotypes. FS-Res animals exhibited a lower percentage of REM sleep compared to susceptible animals at baseline, before FS exposure. In contrast, fourteen days post-stress, resilient animals showed a higher percentage of REM sleep than susceptible animals. This reversal in group ordering between baseline and post-stress conditions is consistent with a stress-dependent reorganization of group differences. Such a crossover pattern may reflect differential sensitivity to stress across groups rather than a uniform effect of stress exposure.

Although we did not test the extinction of aversive context memory, we can hypothesize that the increase in REM sleep of resilient mice could facilitate the learning of the new association between the grid and the safe and secure recording cage context, while reducing emotional reactivity to this object during future re-exposure. This could also explain why resilient mice did not exhibit any defensive behavior towards a novel stimulus. Relevant to this work, it has been shown that an increase in REM sleep following a session of contextual fear extinction facilitates the consolidation of extinction memory in rats (Datta and O’Malley, 2013). Our preclinical mouse model results are consistent with findings in humans, where increasing REM sleep in the second half of the night promotes discrimination between neutral/new and aversive stimuli (Menz et al., 2016) and consolidated REM sleep episodes after trauma appear to be protective against PTSD (Mellman et al., 2007).

One should be more cautious about the role of REM sleep in behavioral resilience: it may represent either a characterization or a consequence of defensive behavior in the present study. Enhancing sleep in stressed animals would help establish a causal link between REM sleep and behavioral resilience to intensive foot-shock stress. However, in support of our hypothesis, the REM sleep increase at day 0 preceded the anxiety-like behavior in a new context (zero-maze) of susceptible animals that did not differ from that of resilient animals at day 4 (data not shown).

Sleep data collected prior to FS exposure revealed pre-existing differences in sleep architecture between groups. At baseline, FS-Res animals spent more time in SWS, while both FS-Esc and FS-Frz animals exhibited increased REM sleep. PTSD-like behavioral severity was positively correlated with REM sleep before FS exposure but negatively correlated with REM sleep 14 days after stress. Together, these findings suggest that baseline sleep architecture—particularly increased REM sleep and reduced SWS—may be associated with vulnerability to maladaptive outcomes following stress, whereas post-stress REM sleep may reflect compensatory processes. Future studies targeting the underlying neural circuits will be required to directly test this hypothesis. Overall, the present study highlights compelling evidence for REM sleep increasing as a marker of resilience after a traumatic stress exposure (Suchecki et al., 2012). Further quantitative (state transitions) and qualitative (EEG spectral analyses) investigations must be carried out to refine sleep phenotyping after high-intensity stress exposure.

To evaluate if our behavioral phenotype analysis was biologically relevant, we assessed c-Fos protein expression as a measure of cellular activation in two main brain areas implicated in emotional processing and defensive behaviors: the amygdala (AMG), and the periaqueductal gray matter (PAG). Consistent with our hypothesis, divergent cellular activities were found between susceptible and resilient mice but also between the two PTSD-like phenotypes after a long-term and full context reminder of the foot-shock procedure. Specific activation of the right part of the AMG corroborated previous findings in PTSD-like memory impairments in mice (Kaouane et al., 2012) suggesting a hypothetical lateralization of emotions in rodents (Gainotti, 2021). Here, c-Fos activation in both the LA and CeL was higher in susceptible mice than in resilient mice 28 days after FS, and 90 min after a full context re-exposure. Our results are in accordance with studies showing an increased number of c-Fos neurons in the CeL and LA of susceptible mice after one week (Skórzewska et al., 2020) or long-term full context recall (An et al., 2021; Milanovic et al., 1998; Silva et al., 2019). Resilient mice showed a higher level of freezing behavior during the context reminder than susceptible freezer mice, but at the same time displayed a lower c-Fos expression in the AMG than susceptible freezers. Notably, the composite score was positively correlated with the number of c-Fos-positive cells in the lateral and central lateral right nuclei of the AMG. In contrast, no such correlation was found between freezing behavior and c-Fos expression in AMG nuclei. Overall, the non-activation of AMG nuclei in this study could suggest a brain inhibitory control mechanism observed in resilient that is impaired in susceptible animals (Wolff et al., 2014). These cellular results reinforce the necessity to study behavior using cluster analysis beyond the basic stressed vs. non-stressed dichotomy. Although PAG subregions show functional specialization—with the posterior PAG mediating freezing and escape, and the anterior PAG linked to fight and avoidance behaviors (Carrive, 1993; Carrive et al., 1997; LeDoux et al., 1988)—their overall connectivity and function remain incompletely understood (Linnman et al., 2012). Analyzing the anterior-to-posterior c-Fos cell expression in the elongated, functionally heterogeneous PAG provides a preliminary view of rostrocaudal differences in activation and connectivity following a foot-shocks exposure. The activation of the ventral PAG in both FS-Frz and FS-Res mice, presenting a high percentage of reminder-associated freezing is consistent with the literature (Brandão and Lovick, 2019; Vianna and Brandão, 2003). Interestingly, PTSD-like phenotypes were distinguished through both the dPAG and the vPAG, but also along the antero-posterior axis. In fact, while FS-Frz presented a high rate of freezing in an FS-associated context and a fewer cellular activation in the anterior part of the dPAG, FS-Esc presented less freezing, more escaping behavior and fewer c-Fos + cells in the posterior part of the dPAG. Furthermore, while both FS-Frz and FS-Res showed high rates of freezing on day 28, they did not present the same cellular activation in the PAG and AMG. The differential neural activity between resilient and freezer mice suggested a distinct freezing behavior and probably separate cognitive processes. It might suggest a dual activation gradient along the antero-posterior and dorsoventral axes of the PAG. Because the central amygdala is connected to both the ventral and dorsal PAG, the internal circuitry involvement could differ between FS-Frz, FS-Esc and FS-Res animals. The differential activation within the dPAG appears to drive the choice between freezing and escaping behaviors. Freezers show reduced anterior dPAG activity, possibly due to inhibitory input, which favors the passive freezing response. Conversely, escapers show reduced posterior dPAG activity, allowing the anterior dPAG to dominate and initiate active flight. Crucially, the AMG is highly activated in both groups, suggesting it provides a uniform high-level threat signal that the dPAG translates into divergent defensive actions. Therefore, dPAG specialization dictates the behavioral output in susceptible animals. In contrast, resilient animals show lower activation in the AMG's lateral/centrolateral nuclei, suggesting they effectively attenuate the initial threat signal at the amygdala, preempting the specialized activation patterns seen in the dPAG. Those possibilities should be tested further using optogenetic control of functional connectivity.

Integrating behavioral, sleep, and neural data highlights how these phenotypes reflect distinct stress-coping strategies. Resilient animals appear to engage adaptive mechanisms, as evidenced by cue-specific defensive responses, increased REM sleep, and selective ventral PAG activation, supporting a role for REM in strengthening relevant memories while weakening irrelevant emotional associations, consistent with the Sleep to Forget, Sleep to Remember (SFSR) theory. Susceptible freezer mice, by contrast, exhibit generalized defensive behaviors, anxiety-like responses, accompanied by heightened amygdala and reduced dorsal PAG activation, suggesting hyperarousal and impaired discrimination between safe and threat cues. The escaper phenotype shows generalized defensive behavior and strong amygdala activation without sleep disruption, indicating engagement of active coping pathways that avoid prolonged hyperarousal. Together, these findings reveal that behavioral expression, sleep regulation, and neural recruitment converge to differentiate coping strategies, highlighting the importance of multi-level analyses in understanding stress susceptibility and resilience. Future studies could further explore how these mechanisms interact to shape long-term stress adaptation in each phenotype.

In conclusion, our analysis of new behavioral phenotypes reveals significant heterogeneity in the behavioral responses of foot-shocked mice, as well as variability in cellular activation within key brain regions implicated in PTSD and associated sleep disturbances. The present findings show the existence of two distinct PTSD-like susceptible phenotypes in mice and suggest a critical role of REM sleep in the behavioral resilience to severe stress. These results pave the way for further preclinical investigations into the behavior and sleep heterogeneity associated with PTSD in murine models.

## CRediT authorship contribution statement

**Emma Lardant:** Data curation, Formal analysis, Investigation, Visualization. **Otilia Kelemen:** Data curation, Formal analysis, Investigation, Visualization. **Louise Pialoux:** Data curation, Formal analysis, Investigation, Visualization. **Coline Gervy:** Data curation, Formal analysis, Software. **Blake Rea:** Investigation. **Betty Poly:** Data curation, Formal analysis, Investigation, Visualization. **Damien Claverie:** Conceptualization, Data curation, Formal analysis, Funding acquisition, Methodology, Project administration, Resources, Supervision, Validation, Visualization, Writing – review & editing. **Frederic Chauveau:** Data curation, Investigation.

## Declaration of competing interest

The authors declare that they have no known competing financial interests or personal relationships that could have appeared to influence the work reported in this paper.

## Figures and Tables

**Supplementary Fig. 2:**
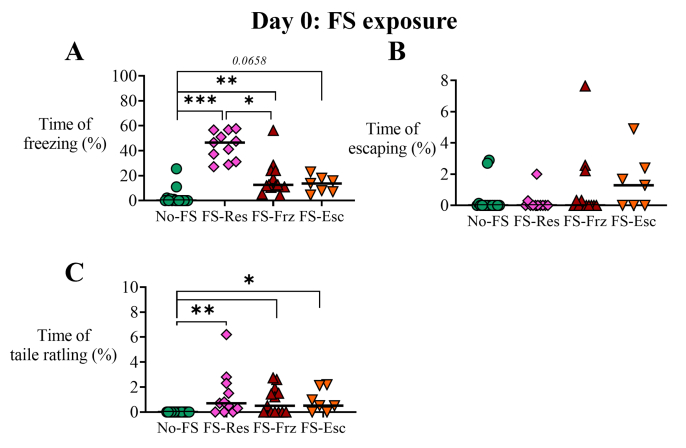
Behavioral responses to foot-shocks A-C. Three defensive responses were assessed during the foot-shock (FS) exposure: the percentage of freezing (A), of escaping (B) and of tail rattling (C). Each symbol stands for an animal: ໐ No-FS, no foot-shocks exposed mice (control), n = 16; ◇FS-Res, foot-shocked resilient mice, n = 11; △FS-Frz, foot-shocked susceptible freezing mice, n = 13 and ▽FS-Esc, foot-shocked susceptible escaping mice, n = 7. Kruskal-Wallis post-hoc analysis: ∗, p-val< 0.05; ∗∗, p-val< 0.01 and ∗∗∗, p-val< 0.001.

## Data Availability

Data will be made available on request.
